# Secondary Resections and Survival After Breast-Conserving Surgery in Breast Cancer Patients: A Cancer Registry-Based Cohort Study

**DOI:** 10.3390/cancers17030369

**Published:** 2025-01-23

**Authors:** Mikhail Ovchinnikov, Alexander Kluttig, Elke Burger, Saskia Thies, Maria Elena Lacruz, Andrea Schmidt-Pokrzywniak, József Mészáros, Holm Eggemann, Atanas Ignatov

**Affiliations:** 1 Department of Plastic, Aesthetic and Hand Surgery, Otto-von-Guericke University, 39120 Magdeburg, Germany; 2 Clinical Cancer Registry Sachsen-Anhalt, 06112 Halle, Germanye.burger@kkr-lsa.de (E.B.); s.thies@kkr-lsa.de (S.T.); e.lacruz@kkr-lsa.de (M.E.L.); andrea.schmidt-pokrzywniak@medizin.uni-halle.de (A.S.-P.); 3 Department of Obstetrics and Gynecology, Otto-von-Guericke University, 39108 Magdeburg, Germany; jozsef.meszaros@med.ovgu.de (J.M.); atanas.ignatov@med.ovgu.de (A.I.); 4 Department of Obstetrics and Gynecology, Clinic Magdeburg, 39108 Magdeburg, Germany; holm.eggemann@klinikum-magdeburg.de

**Keywords:** female breast cancer, breast conservative surgery, re-excision, R1 tumor resection

## Abstract

Breast cancer remains the most common malignancy in women, and breast-conserving surgery (BCS) with adjuvant radiotherapy is a standard treatment for early-stage disease. This study assessed the impact of positive surgical margins and secondary surgeries on local recurrence rates and patient outcomes. In this retrospective tumor registry-based study, 24,450 women were eligible for analysis. Of them, 18,082 underwent primary BCS only, 4836 underwent re-excision by BCS, and 1532 underwent re-excision by mastectomy following primary BCS. Our findings indicate that even though re-excision procedures were associated with higher local recurrence rates (HR 1.19; 95% CI 1.08–1.33), they did not significantly affect long-term survival (HR 0.98; 95% CI 0.87–1.10). This underscores the need for meticulous surgical practices to achieve clean margins initially, and suggests that the choice of surgical intervention should consider individual tumor characteristics and patient preferences to optimize oncological and aesthetic outcomes.

## Simple Summary

Breast cancer is the most common cancer in women, and breast-conserving surgery combined with radiotherapy is a standard treatment for early-stage disease. However, not all surgeries achieve complete removal of the tumor, sometimes requiring additional surgeries. This study analyzed data from over 24,000 women to find out whether it is even necessary to perform a re-resection to achieve clean resection margins and how these secondary surgeries impact recurrence rate and overall survival. The results showed that while additional surgeries slightly increased the risk of recurrence locally, they did not significantly affect long-term survival. These findings highlight the importance of achieving complete tumor removal in the first surgery and suggest that decisions about further surgeries should balance oncological safety with patient quality of life and preferences.

## 1. Introduction

Breast cancer is the most common malignancy among women [1]. The incidence of breast cancer has been steadily increasing [2,3]. Breast-conserving surgery (BCS) followed by adjuvant radiotherapy has become a standard treatment for early-stage breast cancer. This treatment strategy has been extensively compared to mastectomy in terms of patient survival outcomes [4,5,6]. Treatment typically includes surgery, radiation, and systemic therapies, tailored to each patient’s need. Although BCS offers superior cosmetic outcomes and quality of life [7], its limitation lies in the potential for incomplete tumor excision, often necessitating additional surgery such as re-excision or radical mastectomy. Studies have shown a significant increase in local recurrence rates (ipsilateral breast tumor recurrence—IBTR) when tumor cells are present at the resection margin [8,9,10,11,12,13].

Secondary surgeries are associated with higher health risks, increased patient stress, less satisfactory aesthetic results, delays in adjuvant treatment, and elevated healthcare costs [14,15]. The impact of positive surgical margins (R1) on the rate of local recurrence and survival is a topic of ongoing debate. While some studies suggest that adjuvant radiation therapy can control microscopic disease [11], others advocate for re-resection to achieve R0 margins and reduce recurrence risk [16]. The relationship between re-excision after BCS and mortality rate is missing and poorly investigated [17,18]. Consequently, it remains uncertain if the survival benefits observed with BCS also apply to patients undergoing secondary surgery.

This retrospective, tumor registry-based analysis aimed to evaluate the association between re-excision due to positive surgical margins following BCS and both local recurrence rates and overall survival (OS) outcomes.

## 2. Materials and Methods

We investigated female breast cancer cases recorded in the cancer registry of Saxony-Anhalt, a German federal state with a population of approximately 2.2 million inhabitants. This registry systematically records data on diagnosis, age, tumor stage, receptor status, tumor grading, lymph node status, dates of diagnosis, disease recurrence, and death, along with details on treatment regimens employed [19,20]. Our cohort study specifically examined women diagnosed with early breast cancer during the period from 2000 to 2020. A total of 14 hospitals and 13 oncological centers with around 50 physicians are currently participating in the tumor registry.

**Figure 1:**
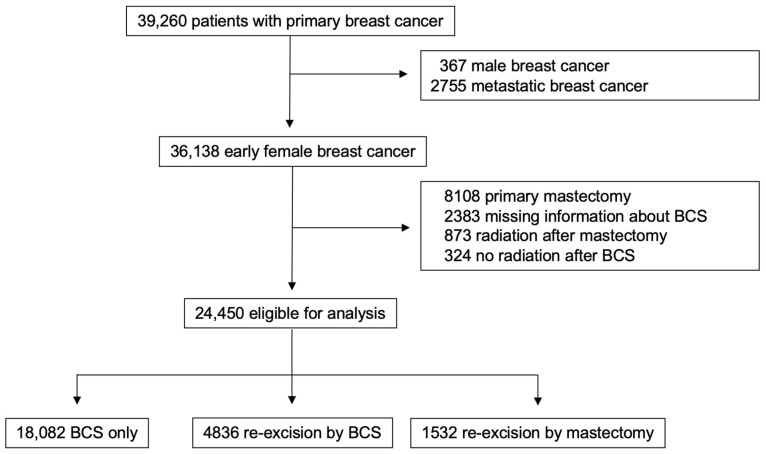
Study design.

The following subgroups of patients were excluded from the initial cohort of 39,260 patients: male breast cancer patients (n = 367), patients with primary metastatic disease (n = 2755), patients who received a primary mastectomy (n = 8108), cases with insufficient reported information regarding breast-conserving surgery (n = 2383), patients who underwent postoperative radiotherapy after mastectomy (n = 873), and patients who did not receive adjuvant radiotherapy after BCS (n = 324). Thus, a total of 24,450 eligible cases were included in this study (Figure 1). They were divided into three groups according to histopathological findings after BCS, as follows:

- Group 1 (BCS only): patients with tumor-free resection margins (n = 18,082, 74%).
- Group 2 (re-excision by BCS): patients who underwent re-excision via BCS because of R1 resection margins (n = 4836, 20%).
- Group 3 (re-excision by mastectomy): patients who underwent re-excision via mastectomy (n = 1532, 6%).

All patients underwent primary surgery within 3 weeks of the date of initial diagnosis, and re-operation within 6 weeks of diagnosis of tumor-positive margins. All patients treated by primary BCS only or re-excision by BCS were treated subsequently with adjuvant radiotherapy within 12 weeks. None of the patients in this study died between the date of diagnosis and initial surgery.

The primary outcome was disease-free survival (DFS), defined as the interval between the date of diagnosis and the occurrence of either local or regional recurrence, distant metastasis, or death, whichever occurred first. The secondary outcome was overall survival (OS), which was the time from the date of diagnosis to death by any cause. The follow-up ended with either the patient’s death, the date of last available information, or the last follow-up on 31 December 2019.

### Statistical Methods

Statistical calculations were performed using SPSS version 28.0 (SPSS, IBM Corp., Armonk, NY, USA). Clinical, pathological, and treatment parameters between groups were compared using the chi-squared test or Fisher’s exact test for categorical variables, and the two-sample *t*-test for continuous variables. Survival probabilities were estimated via the Kaplan–Meier method. The equality of survival curves was tested by the log-rank test [21]. Statistical analyses were two-sided, and *p*-values of less than 0.05 were statistically significant. To keep the influence of confounding bias to a minimum, we performed an adjusted Cox regression analysis.

## 3. Results

**Figure 2:**
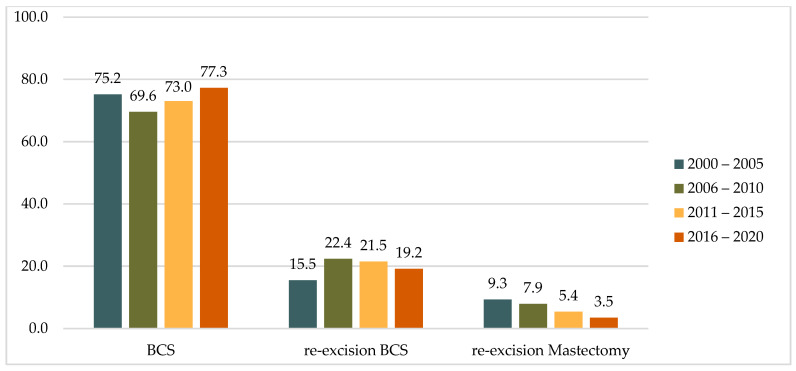
Distribution of operations in five-year intervals, (%).

A comparison of the two study decades (2000–2010 vs. 2011–2020) revealed an increase in the proportion of primary BCS cases from 71.9% to 75.1%. During the same periods, there was a 48% decrease in the rate of mastectomies after the detection of positive resection margins after primary BCS, from 8.5% to 4.5%, while the rate of re-excisions by BCS remained almost unchanged at 19.7% and 20.4%, respectively. A similar trend was observed across four five-year intervals (2000–2005, 2006–2010, 2011–2015, and 2016–2020), with the rate of mastectomy as a re-operation decreasing steadily from 9.3% to 3.5% (Table 1 and Figure 2).

The median age at diagnosis was 61 years (range 19–102 years) for the BCS only group, 59 years (range 23–94 years) for re-excision by BCS, and 62 years (range 29–91 years) for the group of patients treated by mastectomy as re-excision. Invasive carcinoma of no special type (NST) was the predominant histological subtype across all three groups, accounting for 82.1%, 81.2%, and 71.9% of cases, respectively (Table 2). Lobular cancer was found significantly more often in the group with re-excision by mastectomy (20.9%) than it was in the BCS only group and the re-excision by BCS group (10.2% and 11.5%, respectively).

Low-grade tumors (G1) were identified more often in the first group treated by BCS only (17.2%). In the groups treated by BCS and mastectomy as re-excision procedures, the rates of G1 tumors were 15.8% and 10.6%, respectively.

Most patients in all groups had tumor sizes of T1 or T2. Tumors up to 20 mm in size were expectedly more frequent in the group treated by BCS only (64.1%) and less frequent in the group treated by re-excision mastectomy (55.5%). In this regard, patients with larger tumors (T3 and T4) predominated in the third group.

Also as expected, the absence of metastases to regional lymph nodes was predominant in the group of patients treated by BCS only (75.6%), while in groups 2 and 3 negative lymph node statuses were 66.4% and 70.1%, respectively. The presence of lympho-vascular invasion (LVSI) was detected more frequently in the groups with re-operation by BCS and by mastectomy (40.1% and 42.4%, respectively). In the BCS only group, LVSI was observed in only 32.8% of cases.

The majority of tumors across all groups were hormone receptor-positive (*p* = 0.035), with no significant differences observed between groups (Table 2). Hormone receptor-negative status, however, was associated with reduced recurrence-free survival (HR = 1.33; *p* < 0.001) and overall survival (HR = 1.45; *p* < 0.001). These findings highlight the critical role of hormonal status in guiding treatment decisions and predicting patient outcomes.

Most tumors in all groups were hormone receptor-positive. HER2 positivity was observed in 15.6% of the BCS only group, and in 19.1% and 19.8% of re-excision groups by BCS and mastectomy, respectively.

Rates of local–regional recurrence were further analyzed and compared across groups. As shown in Table 3, the highest local recurrence rate (6.6%) was observed in patients undergoing re-excision via BCS. In contrast, in the group of patients treated by BCS only and the re-excision by mastectomy group, local recurrence rates were 4.0% and 2.3%, respectively. Regional recurrence was least frequent in the group of patients who underwent re-excision by BCS (0.9%), in contrast to the BCS only group (1.1%) and the re-excision by mastectomy group (2.0%).

Next, we evaluated the impact of the type of surgery on survival outcome. The five-year DFS in the BCS group was 64.9%, in the re-excision by BCS group it was 63.3%, and in the re-excision by mastectomy group, 70.6% (*p* < 0.001). Five-year OS was 62.2% for the BCS only group, 67.7% for the re-excision BCS group, and 73.5% for the re-excision mastectomy group.

**Figure 3:**
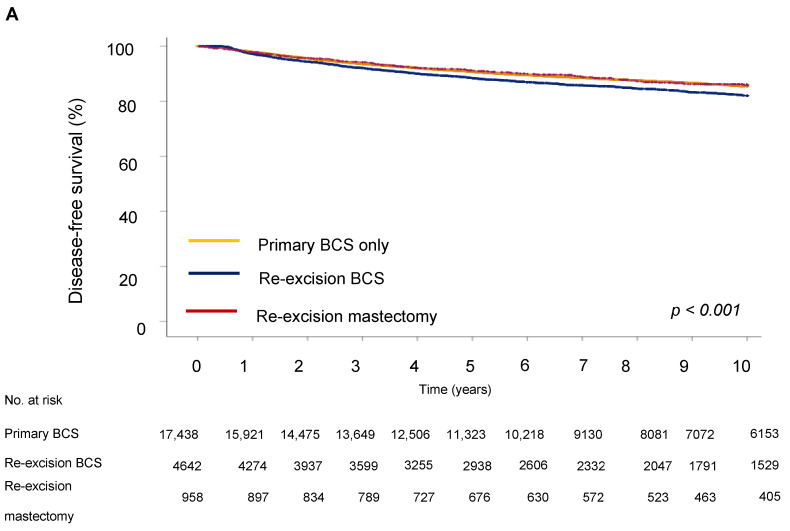

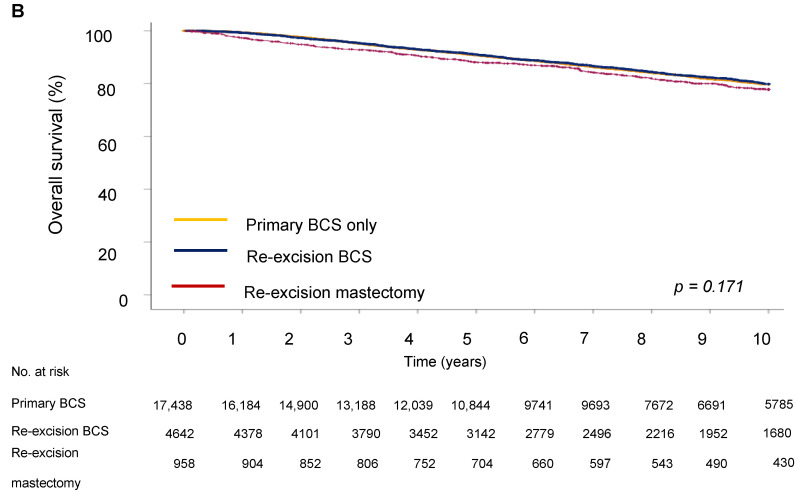
Survival outcomes by surgery type. (**A**) Disease-free and (**B**) overall survival of breast cancer patients.

For disease-free survival (DFS), re-resection by BCS was associated with an increased risk of recurrence (HR = 1.19; 95% CI 1.08–1.33) compared to BCS only (Table 4 and Figure 3). However, the type of surgery was not significantly associated with OS, showing no notable differences between groups (HR = 0.98; 95% CI 0.87–1.10), indicating that the effect of surgery type on long-term survival could be due to other tumor characteristics, such as proliferation activity, aggressiveness, hormone receptor status, and following adjuvant treatment. Analysis showed that a high grade of tumor differentiation (G3) was associated with an increased risk of recurrence (HR = 1.69, 95% CI, *p* < 0.001 for DFS and HR = 1.59, 95% CI, *p* < 0.001 for OS). T2 and T3 tumor sizes correlated with reduced DFS (HR = 1.72, 95% CI, *p* < 0.001) and OS (HR = 1.72, 95% CI, *p* < 0.001). Lymph node metastases at initial diagnosis were associated with significantly reduced DFS (HR = 1.77 (1.60–1.96), 95% CI, *p* < 0.001) and OS (HR = 1.92 (1.73–2.14), 95% CI, *p* < 0.001), emphasizing the role of lymphatic invasion in disease progression. Positive HER2 expression was associated with an increased risk of recurrence (HR = 1.87, 95% CI, *p* < 0.001 for DFS) and decreased OS (HR = 1.77, 95% CI, *p* < 0.001).

## 4. Discussion and Conclusions

The findings of our study provide insight into the implications of positive resection margins in breast cancer patients undergoing BCS, and particularly their impact on disease-free and overall survival. The stepwise five-year analysis reflects a trend toward an increasing rate of primary BCS and a decreasing rate of re-resections at later stages. This trend likely reflects advancements in early diagnosis, surgical techniques, individualized treatment approaches, and the development of non-surgical therapies, which have ultimately reduced the need for more radical procedures such as mastectomy.

The first important aspect of our study was to identify the frequency of local–regional recurrences after BCS depending on the status of the resection margin. Consistent with earlier findings by Houssami et al. [22], our study revealed a higher incidence of local recurrence in patients with positive resection margins compared to those with negative margins. This aligns with the established understanding that complete tumor resection with no residual cancer cells at the margin (R0) is critical in minimizing the risk of local recurrence [23]. Despite the clear statistical association between positive resection margins and increased recurrence rates, the causality behind this relationship remains complex and not straightforward. Currently, there is no consensus on the state of resection margins in the context of further surgical treatment of breast cancer. This concerns both the acceptable minimum distance from the tumor margin and the need for re-operation. The issue of intraoperative examination of resection margins also remains controversial. According to M. Silverstein et al. [24], increasing the width of resection margins from 1 to 10 mm leads to a nearly fivefold decrease in local recurrence rates (from 42% to 8.3%). It is also stated that at a sufficient resection width (10 mm or more) from the tumor margin practically does not influence the risk of local recurrence. The increased recurrence risk in cases of positive resection margins may be attributed to the intrinsic biology of the tumor, predisposing it to more aggressive behavior, rather than just the margin status itself [25]. We should also be mindful of differences in national standards and guidelines for the acceptable width from the resection margin between the early 2000s and the present day. Within breast-conserving surgery, the standards and amount of tissue resected varied significantly among individual surgeons, centers, and even countries. The lack of consensus is evident in various articles and consensus papers from the first decade of this century [26,27,28,29]. Information on acceptable resection margins is also missing in the early versions of S3 Leitlinien and NCCN Guidelines, while the latest version 4.2024 recommends the principle of “no ink on tumor” for invasive breast cancer and ductal carcinoma in situ (DCIS), invasive cancer with an extensive DCIS component, or invasive cancer treated with neoadjuvant chemotherapy followed by BCS. A minimum of 2 mm from the resection margin is required for pure DCIS and DCIS with microinvasion to ensure the radicality of the operation [30]. According to a survey, 35% of surgeons would opt for re-excision if the tumor was within 1 mm of the anterior margin, and 10% would excise the margins again if the tumor was located less than 1 mm from the posterior margin [31]. Further study has shown that with a width from the resection margin of less than 1 mm, and if tumor cells are stained at the resection margin, additional boost therapy to the tumor bed may be necessary. Local recurrences were exceedingly rare in cases where stained tumor cells were found. Typically, patients with positive margins underwent re-excision to achieve clear margins [32]. In most cases, no residual tumor was detected in the specimen following re-excision or even mastectomy [33]. The frequency of tumor cell detection during re-excision did not exceed 38% [34]. There are also data indicating that wider resection margins only slightly reduce the rate of IBTR compared with the “no ink on tumor” principle and do not significantly affect IBRT rates in younger patients or those with unfavorable biology, lobular cancer, or cancer with an extensive intraductal component [35]. However, a significant disadvantage of extensive resection or re-resection of the margins is the possibility of a visible cosmetic defect, which can greatly impact the psychological well-being of patients [36].

Our findings demonstrate significant differences in age at diagnosis, histological type, tumor grade, size, lymph node involvement, lympho-vascular invasion, and hormonal/HER2 receptor status across patient groups. These factors are critical for tailoring treatment strategies and predicting disease outcomes. Thus, HER2 expression was associated with an increased risk of recurrence and decreased OS, which is consistent with the known aggressiveness of HER2-positive tumors. The presence of LVSI also significantly worsened the prognosis of DFS (HR = 1.98, 95% CI) and OS (HR = 2.03, 95% CI), indicating its importance both as an indicator of disease progression and as a potential target for therapeutic intervention. In the context of OS, differences between the groups were not statistically significant, which may indicate that the long-term survival of patients is determined not only by the choice of surgical strategy, but also by other factors, such as the effectiveness of subsequent treatment, including chemotherapy, hormonal therapy, targeted therapy, and radiotherapy.

G3 tumor differentiation was associated with an increased risk of recurrence, confirming the significance of tumor grading as a prognostic indicator and highlighting the critical importance of early detection of disease. A patient’s age at diagnosis of early breast cancer, however, had no significant effect on recurrence-free survival, but it was a significant factor for overall survival. This may reflect a more aggressive disease course in younger patients or differences in treatment efficacy. This complexity underscores the importance of considering individual patient and tumor characteristics in surgical decision-making.

Another key aspect of our study was to assess whether re-resection to achieve R0 margins is necessary in cases initially identified with R1 margins. Our results suggest that, while achieving R0 margins is preferable, the decision for re-resection should be individualized, considering factors such as tumor biology, patient age, comorbidities, and quality of life. This is in line with the perspective of Azu et al. [37], who advocate for a more personalized approach to managing R1 margins. Factors such as tumor biology, patient age, comorbidities, and the potential impact on quality of life should be weighed against the benefits of re-resection. Studies have shown that adjuvant radiation and systemic therapies can significantly mitigate the risk associated with R1 margins [38,39]. Therefore, the decision for re-resection must be integrated within the broader context of a multidisciplinary treatment plan. The relationship between re-excision and local–regional recurrence has been studied in more detail, although with mixed results. Some studies have associated re-excision with an increased risk of recurrence [18], while others have found no such association [40]. This discrepancy may be due to residual deterrence, emphasizing the need for further investigation of whether re-excision is sometimes unwarranted. Our results suggest that in a broader population context, re-excision, regardless of method, has a significant impact on local recurrence rates and, to a lesser extent, on overall survival rates. Previous studies of BCS with and without re-excision also reported similar survival outcomes, but comparisons with primary mastectomy treatment have been limited. Another comprehensive study conducted in Denmark showed similar overall survival rates among patients who underwent both BCS and oncoplastic surgery [41], which once again reminds us of the need for a multidisciplinary approach and the importance of informing patients before starting treatment about the possibility of oncoplastic resections or reconstructive surgery if a decision is made in favor of more radical surgical treatment. This is a very important psychosocial aspect, especially in young patients, including body perception, sexuality, and psychological well-being. In this context, data on recurrence rates and disease-free survival in the group of patients who underwent mastectomy with one-stage or delayed reconstruction as a re-operation would be of particular interest, which may be the subject of future studies. Furthermore, the higher frequency of re-excisions as mastectomies in lobular cancer cases suggests the need for research into upfront mastectomy in selected cases. The healthcare burden from multiple BCS procedures due to positive margins and recurrences should be further explored to optimize surgical strategies in terms of resource use.

Our study was the first of its kind to use a large patient cohort to combine an analysis of recurrence development after BCS and re-operations and to examine the relationship between DFS and OS rates after different types of surgeries. The results of our study support the hypothesis that the type of surgery has a significant effect on the likelihood of local–regional recurrence. The increased DFS interval after mastectomy may be due to a more radical removal of tissue, whereas BCS re-resection may be associated with a higher risk of leaving microscopic foci of cancer. These data suggest that mastectomy may be more effective in preventing recurrence compared with BCS. However, despite the increased local recurrence rates after re-resections, the long-term overall survival of patients remained largely unaffected, suggesting the need to better explore mechanisms that may explain these differences in recurrence rates. Individual tumor characteristics and patient preferences, especially in the balance between quality of life after surgery and the risk of disease recurrence, should be taken into an account when choosing a surgical method in order to optimize both oncological and aesthetic results. Thus, this analysis provides important data for clinicians and researchers aimed at optimizing surgical treatment and improving the prognosis for patients with local recurrences.

### Strengths and Limitations

This study had several strengths, including a large sample size of 23,380 cases from a population-based cancer registry, offering robust statistical power and generalizability. The long follow-up period of up to 20 years provided valuable insights into long-term prognosis, while the use of multivariate analysis helped control for confounding variables. Furthermore, real-world data from a cancer registry ensure that these results reflect actual clinical practices.

However, there are limitations to consider. Registry data may suffer from underreporting, particularly regarding recurrence events, potentially leading to an underestimation of recurrence rates. Additionally, surgical techniques and margin assessment standards have evolved over the two decades and through different centers covered by this study, introducing variability that could affect the results.

## Figures and Tables

**Table 1:** Distribution of operations in five-year and ten-year intervals.

In %		2000–2010	2011–2020		
	Primary BCS only	71.9	75.1		
	Re-excision BCS	19.7	20.4		
	Re-excision Mastectomy	8.5	4.5		
In %		2000–2005	2006–2010	2011–2015	2016–2020
	Primary BCS only	75.2	69.6	73	77.3
	Re-excision BCS	15.5	22.4	21.5	19.2
	Re-excision Mastectomy	9.3	7.9	5.4	3.5

**Table 2:** Patient and tumor characteristics.

Variable	Primary BCS Only	Re-Excision by BCS	Re-Excision by Mastectomy	*p*-Value
Age at diagnosis of metastatic disease, median, y				<0.001
	61 (19–102)	59 (23–94)	62 (29–91)	
Histology				<0.001
NST	14,845 (82.1%)	3927 (81.2%)	1101 (71.9%)	
Lobular	1852 (10.2%)	5578 (11.5%)	320 (20.9%)	
Others	1385 (7.7%)	352 (7.3%)	111 (7.2%)	
Grade, n (%)				<0.001
1	3028 (17.2%)	744 (15.8%)	158 (10.6%)	
2	9937 (56.3%)	2788 (59.1%)	907 (61.0%)	
3	4683 (26.5%)	1185 (25.1%)	421 (28.3%)	
T status at initial diagnosis, n (%)				<0.001
T1	10,902 (64.1%)	2877 (63.1%)	517 (55.5%)	
T2	5499 (32.3%)	1532 (33.6%)	363 (38.9%)	
T3	330 (1.9%)	102 (2.2%)	40 (4.3%)	
T4	272 (1.6%)	48 (1.1%)	12 (1.3%)	
Lymph node metastases, n (%)				<0.001
Negative	12,970 (75.6%)	3048 (66.4%)	662 (70.1%)	
Positive	4187 (24.4%)	1543 (33.6%)	282 (29.9%)	
LVSI, n (%)				<0.001
Negative	8885 (67.2%)	2111 (59.9%)	398 (57.3%)	
Positive	4331 (32.8%)	1413 (40.1%)	294 (42.4%)	
Hormone receptor, n (%)				0.035
Negative	3035 (18.4%)	899 (20.1%)	172 (19.2%)	
Positive	13,467 (81.6%)	3577 (79.9%)	725 (80.8%)	
HER2 receptor, n (%)				<0.001
Negative	5745 (38.3%)	1629 (39.2%)	290 (37.1%)	
Low	6901 (46.0%)	1732 (41.7%)	337 (43.1%)	
Positive	2343 (15.6%)	791 (19.1%)	155 (19.8%)	

**Table 3:** Recurrence characteristics.

Local–Regional Recurrence	Primary BCS Only n = 18,082	Re-Excision by BCS n = 4836	Re-Excision by Mastectomy n = 1532
No.	17,069 (94.4%)	4420 (91.4%)	1464 (95.6%)
Local	723 (4.0%)	319 (6.6%)	35 (2.3%)
Regional	199 (1.1%)	44 (0.9%)	31 (2.0%)
Both	91 (0.5%)	53 (1.1%)	2 (0.1%)

**Table 4:** Multivariate analysis of DFS and OS.

Variable	DFS		OS	
	HR (95% CI)	*p*-Value	HR (95% CI)	*p*-Value
Surgery BCS only Re-excision by BCS	Reference 1.19 (1.08–1.33)	0.001	Reference 0.98 (0.87–1.10)	0.670
Age	0.96 (0.86–1.06)	0.431	1.96 (1.76–2.17)	<0.001
Grading G1/2 G3	Reference 1.69 (1.52–1.87)	<0.001	Reference 1.59 (1.43–1.77)	<0.001
Stage T1 T2,3	Reference 1.72 (1.56–1.91)	<0.001	Reference 1.72 (1.55–1.90)	<0.001
Lymph node status Negative Positive	Reference 1.77 (1.60–1.96)	<0.001	Reference 1.92 (1.73–2.14)	<0.001
HER2 Negative Positive	Reference 1.14 (1.01–1.28)	0.032	Reference 1.00 (0.88–1.15)	0.978
Adjuvant chemotherapy None Yes	Reference 1.47 (1.28–1.68)	<0.001	Reference 0.79 (0.70–0.89)	<0.001

## Data Availability

Restrictions apply to the availability of these data. Data were obtained from Klinische Krebsregister Sachsen-Anhalt and are available at https://www.kkr-lsa.de/ with the permission of Klinische Krebsregister Sachsen-Anhalt.
